# Electrical Stimulation for Wound-Healing: Simulation on the Effect of Electrode Configurations

**DOI:** 10.1155/2017/5289041

**Published:** 2017-04-09

**Authors:** Yung-Shin Sun

**Affiliations:** Department of Physics, Fu Jen Catholic University, New Taipei City 24205, Taiwan

## Abstract

Endogenous electric field is known to play important roles in the wound-healing process, mainly through its effects on protein synthesis and cell migration. Many clinical studies have demonstrated that electrical stimulation (ES) with steady direct currents is beneficial to accelerating wound-healing, even though the underlying mechanisms remain unclear. In the present study, a three-dimensional finite element wound model was built to optimize the electrode configuration in ES. Four layers of the skin, stratum corneum, epidermis, dermis, and subcutis, with defined thickness and electrical properties were modeled. The main goal was to evaluate the distributions of exogenous electric fields delivered with direct current (DC) stimulation using different electrode configurations such as sizes and positions. Based on the results, some guidelines were obtained in designing the electrode configuration for applications of clinical ES.

## 1. Introduction

A wound is a type of injury in which the skin epithelial layer is broken [1], and the break may go beyond the skin epidermis to deeper layers such as the dermis, the subcutis, and muscle. In 1843, Du Bois-Reymond first measured an electric current of 1 *μ*A flowing out of a cut on his finger [2, 3], and more recently, currents of 35 and 10~30 *μ*A/cm^2^ were recorded in the amputated fingers of children and the wounds of guinea pigs, respectively [4]. Since then, it has been demonstrated that endogenous DC electric fields (EFs) occur naturally, in vivo around wounds. An electric potential difference of 30~100 mV, known as the transepithelial potential (TEP), was measured between the epidermis and the dermis in the normal skin of a cavy [4]. Nuccitelli et al. reported that the mean lateral electric field in the space between the epidermis and stratum corneum adjacent to a lancet wound was around 100~200 mV/mm, and this value was largest in fresh wounds and slowly declined as the wound closed [5]. The TEP is known to occur as a result of the accumulations of negative and positive charges on the surface of and inside the epidermis, respectively. When the ion channels of Na^+^, K^+^, and Na^+^/K^+^ ATPase distribute unevenly in the apical membrane of the skin's mucosal surface, a transepithelial potential difference (TEPD) is established [6, 7]. And once the epidermis is broken, an electrical leak is produced since the resistance of the wounded site is lower than that of the normal skin. An endogenous EF is then created by reason of the net movement of ions within the layer between the epidermis and the dermis.

This endogenous EF is highly involved in the wound-healing process mainly through its effects on protein synthesis and cell migration [8–10]. G. J. Bourguignon and L. Y. W. Bourguignon exposed human fibroblasts to high voltage, pulsed current stimulation (HVPCS) to increase the healing rate of soft tissue injuries [11]. It was found that the rates of both protein and DNA syntheses were significantly increased by specific combinations of HVPCS voltage and pulse rate; the optimal EFs of protein and DNA syntheses were measured to be 6.7 and 10 V/cm, respectively, with a pulse rate of 100 pulses/sec and the cells located near the negative electrode, and HVPCS intensities greater than 250 V, corresponding to EFs higher than 33.3 V/cm, inhibited both protein and DNA syntheses. Moreover, it has long been proposed that, near the wounded area, cells migrate in response to the endogenous EF to repair the wound. This phenomenon, known as electrotaxis or galvanotaxis, describes the directional migration of cells toward the cathode or anode of an applied EF. There are three sequential, distinct, but overlapped phases involved in a normal wound-healing process: the inflammatory, the proliferation, and the remodeling phases. During the inflammatory phase, the endogenous EF enhances autolysis and phagocytosis by means of the electrotaxis of macrophages and neutrophils. Investigators have shown that macrophages exposed to a 1 Hz and 2 V/cm EF exhibited an induced migration velocity of around 5.2 × 10^−2 ^*μ*m/min on a glass substrate, possibly due to EF exposure inducing the reorganization of microfilaments from ring-like structures to podosomes [12]. Also, Kindzelskii and Petty reported that the application of extremely low-frequency pulsed DCEFs that were frequency- and phase-matched with endogenous metabolic oscillations led to greatly exaggerated neutrophil extension and metabolic resonance [13]. As the proliferative phase begins, EF promotes fibroplasia by guiding fibroblasts toward the wounded area. Guo et al. demonstrated that human dermal fibroblasts of both primary and cell-line cultures migrated directionally toward the anode in an EF of 50~100 mV/mm [14]. Chao et al. applied static and pulsed DCEFs to calf anterior cruciate ligament (ACL) fibroblasts and found that these cells showed enhanced migration speed and perpendicular alignment to the applied EFs [15]. In the remodeling phase, EF accelerates wound contraction and epithelialization by directing the migration of myofibroblasts, keratinocytes, and epidermal cells near the wounded area. Nishimura et al. found that primary human keratinocytes migrated randomly on collagen substrates in EFs of 5 mV/mm or less, but in fields greater than 50 mV/mm they migrated toward the cathode pole of the field [16]. Cooper and Schliwa showed that, in EFs of 0.5~15 V/cm, single epidermal cells, cell clusters, and cell sheets migrated toward the cathode, with clusters and sheets breaking apart into single migratory cells in the upper range of these field strengths [17].

Knowing that the endogenous EF is highly involved in the wound-healing process, researchers have also demonstrated that exogenous EFs are beneficial for the healing of wounds in both animal and human models [18–24]. Electrotherapies including microcurrent electrical stimulation (MES) [25] and transcutaneous electrical nerve stimulation (TENS) [26] have become a current trend in wound-healing applications. Depending on the type of currents, ES can be divided into three categories: DC, alternating current (AC), and pulsed current (PC) [8, 27, 28]. In 1968, DC was first applied on human wounds, showing that patients with chronic leg ulcers healed after 50~100 *μ*A DC treatments for six weeks [29]. Carley and Wainapel observed that low intensity direct current (LIDC) in the range of 200 to 800 *μ*A yielded 1.5 to 2.5 times faster wound-healing rates in thirty patients with indolent ulcers located either below the knee or in the sacral area [18]. Symmetric square wave (an AC form) and asymmetric biphasic pulsed wave (a PC form) were applied on wounds. Baker et al. evaluated the effects of these two stimulation waveforms on healing rates in patients with diabetes and open ulcers. They found that electrical stimulation, given daily with a short pulsed, asymmetric biphasic waveform, enhanced healing by nearly 60% over the control rate of healing [21]. Electrical nerve stimulation (ENS) was applied to patients with stasis ulcers, indicating an increase in the healing rate from 15% (sham treatment) to 42% after 12 weeks of treatment [30]. Moreover, low voltage PC (LVPC) [31, 32] and high voltage PC (HVPC) [33–35] electrotherapies have been shown to accelerate wound-healing in many clinical studies. For example, doubled-peaked monophasic impulses of a total duration of 0.1 ms, frequency of 100 Hz, and amplitude of 100 V were demonstrated to be an efficient method for better healing of crural ulceration [36].

The present study was aimed to optimize the DC stimulation therapy for wound-healing enhancement. Given that the endogenous EF is beneficial and necessary for wound-healing, it is important to determine the parameters in exogenous electrotherapy. We built a three-dimensional wound model consisting of different tissue types in the skin layers. Using the finite element method (FEM) and the commercial software COMSOL Multiphysics, we simulated the distribution of EF near the wounded area under different electrode configurations and further compared these results with what were observed in the endogenous case. The main goal of this study was to evaluate the effects of electrode configurations, including sizes and positions, on the EFs produced around the wound. The total power dissipation due to Joule heating in different skin layers was also discussed.

## 2. Materials and Methods

Within the biological tissue, the electric field resulting from constant DC can be treated as quasi-stationary over time. Steady EFs are established by flowing constant DC through volume conductors with homogeneous and isotropic electrical properties such as conductivity and relative permittivity. Under this circumstance, the distribution of electric potential (*V*) is governed by the Laplace equation, ∇^2^*V* = 0, with appropriate boundary conditions. In the Dirichlet boundary condition, a fixed scalar potential, the applied voltage, is specified on the surface of the model.

### 2.1. The Wound Model

**Figure 1:**
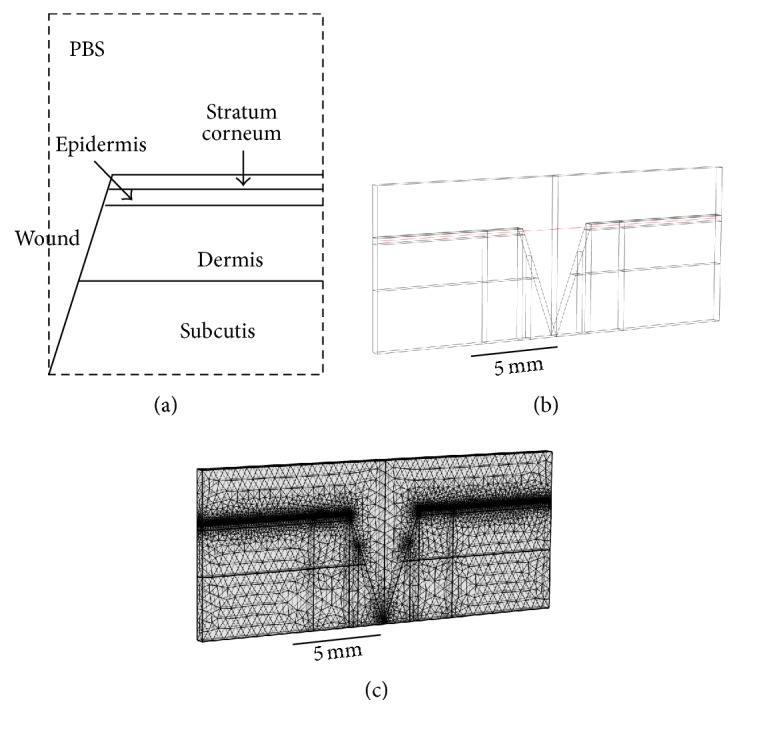
(a) The geometry of the wound and the skin (not to scale). (b) The wound model constructed in COMSOL. (c) The finite element mesh constructed in COMSOL.

A three-dimensional finite element wound model was built using the software COMSOL Multiphysics (MI, USA). The geometry of the wound and the skin is shown in Figure 1(a) with dimensions listed in Table 1. The outermost layer of the epidermis, the stratum corneum, has a thickness of 0.014 mm and is composed of 15~20 layers of flattened cells. The epidermis, having a thickness of 0.3 mm, is composed of proliferating basal and differentiated suprabasal keratinocytes. The dermis, consisting of connective tissues, has a thickness of 2.2 mm. The underlying subcutis (also called the subcutaneous tissue or the hypodermis), with a thickness of 3 mm, has three types of cells: fibroblasts, adipose cells, and macrophages. The wound and the surrounding tissue were immersed in a salty buffer, phosphate-buffered saline or PBS, for better electrical conductance.  Figure 1(b) shows the wound model constructed in COMSOL. Viewing from top, the wound and the skin were modeled as a cylinder with a total thickness of 5.514 mm, and the wound itself had a side view of a triangle with a base of 4 mm and a height of 5.514 mm (see Figure 1(b)).  Figure 1(c) shows the finite element mesh made of 485,510 tetrahedral elements, 73,114 triangular elements, 3,662 edge elements, and 92 vertex elements.

### 2.2. Tissue Properties

The electrical properties of different skin layers are listed in Table 1 [37–41]. For simplicity, the four skin layers were modeled as homogenous, isotropic conductors with constant conductivities and relative permittivities throughout. The conductivities of the stratum corneum, the epidermis, the dermis, and the subcutis were 2 × 10^−6^, 0.026, 0.222, and 0.08 Sm^−1^, respectively. The relative permittivities of these four tissues were 5 × 10^2^, 10^6^, 10^8^, and 10^7^, respectively. The conductivity and relative permittivity of the surrounding PBS buffer were 1.4 Sm^−1^ and 80, respectively. We neglected the facts that epidermis and dermis are polarized epithelia and there are numerous Na^+^/K^+^ pumps on the membranes of each layer. In other words, we were most interested in optimizing the electrode configurations when applying external ES. A more detailed model including polarized epithelia and ion pumps is required if one wants to elucidate the underlying mechanism of the endogenous EF.

### 2.3. Simulation Conditions

**Figure 2:**
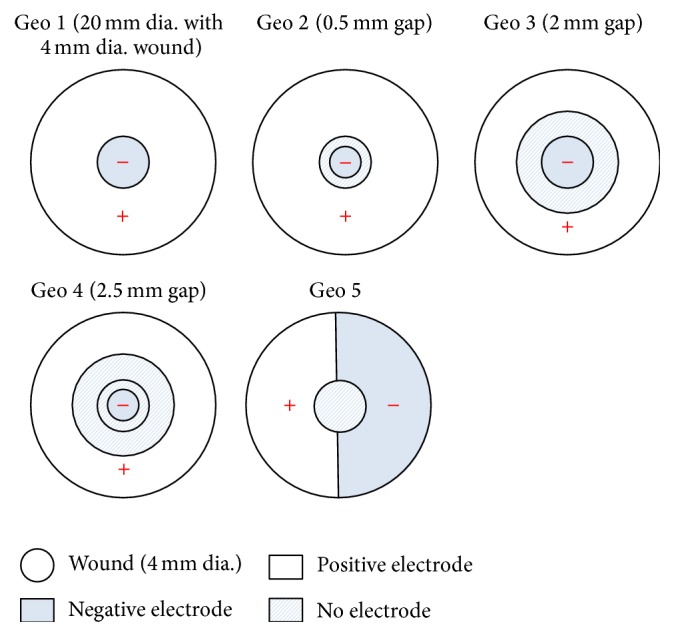
Five electrode configurations used in this study.

The wound model was first used to simulate the distribution of endogenous EF around the wounded area. A potential difference of 30 mV was established between the top of the stratum corneum and the bottom of the epidermis. The ground (0 V) was set on the surface of uninjured skin and a potential of 30 mV was placed on the interface between epidermis and dermis. Further, this model was applied to studying the effects of arrangements and sizes of electrodes on the distribution of exogenous EFs. We would like to find the optimal electrode configuration that has a synergistic effect to the existing endogenous EF. The top view of the five electrode configurations is shown in Figure 2. In the first configuration (Geo 1), the diameter of the circular, negative electrode was 4 mm. This electrode covered the whole wounded area and was grounded at 0 mV. The remaining intact skin was covered with the positive electrode assigned an electric potential of 30 mV. In the second configuration (Geo 2), the negative electrode, with a diameter of 3 mm, was placed on the center of the wound. There was a ring-shaped gap of 0.5 mm between the positive (30 mV) and negative (grounded) electrodes. In the third configuration (Geo 3), the negative electrode covered the whole wounded area (diameter = 4 mm), and there was a ring-shaped gap of 2 mm between it (grounded) and the positive (30 mV) electrode. In the fourth configuration (Geo 4), the positive (30 mV) and negative (grounded) electrodes partially covered the intact skin and the wounded area, respectively, and there was a ring-shaped gap of 2.5 mm in between. In the last configuration (Geo 5), no electrodes were placed on the wound, and the positive (30 mV) and negative (grounded) electrodes covered each side of the intact skin.

## 3. Results and Discussion

**Figure 3:**
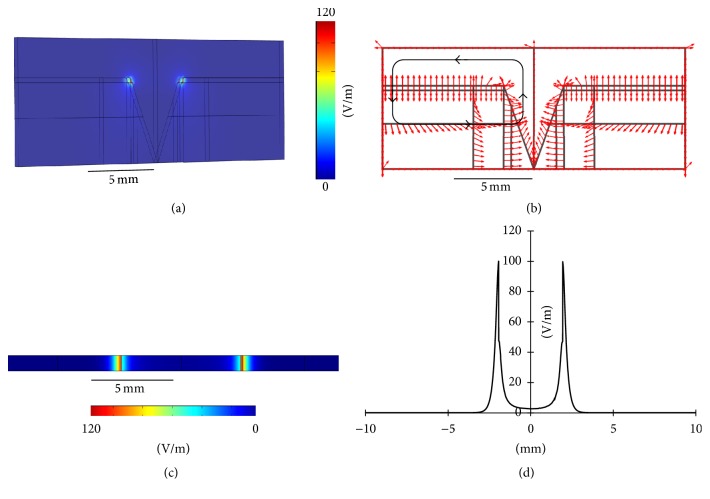
(a) 3D distribution of the endogenous EF. (b) Direction of the electric current flow. (c) 2D distribution of the endogenous EF. (d) 1D distribution of the endogenous EF.

The endogenous EF due to a potential difference of 30 mV between the top of the stratum corneum and the bottom of the epidermis is shown in Figure 3. The EF strength near the edge of the wound (i.e., the junction of the wound and the intact skin) was close to the theoretical value of 30 mV/0.314 mm, or 96 mV/mm, as indicated three-dimensionally (3D) in Figure 3(a).  Figure 3(b) shows the direction of the electric current flow, indicating the formation of a current loop (marked as a black loop with arrows). In Figure 3(c), the two-dimensional (2D) EF distribution, taken along the horizontal plane marked red in Figure 1(b) (the middle of the epidermis layer), shows an EF value of around 96 mV/mm near the edge of the wound. In Figure 3(d), the one-dimensional (1D) EF distribution, plotted along the horizontal line marked red in Figure 1(b) (taken as the *x*-axis from 0 to 20 mm), further verifies that the EF distributes near the wound edge (*x* = 8 mm and *x* = 12 mm) and drops to zero outward along the intact skin (*x* < 8 mm and *x* > 12 mm).

**Figure 4:**
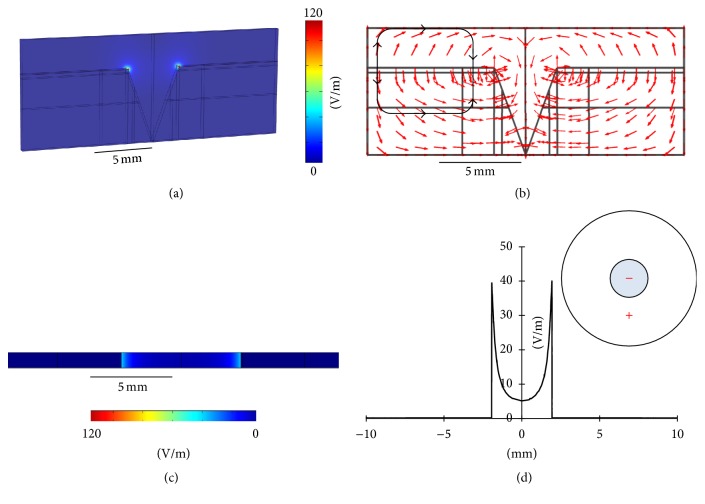
(a) 3D exogenous EF distribution in Geo 1. (b) Direction of the electric current flow in Geo 1. (c) 2D exogenous EF distribution in Geo 1. (d) 1D exogenous EF distribution in Geo 1.

Figure 4(a) represents the 3D exogenous EF distribution in the Geo 1 configuration. Similarly, the EF strength had a maximum near the edge of the wound, and this value decreased sharply to almost zero right out of the wound toward the intact skin. The direction of the electric current flow, as shown in Figure 4(b), indicates a clockwise current flow outside the skin (the top part of the black loop) and a counterclockwise current flow inside the skin (the top part of the black loop). It is the counterclockwise current that could help in wound-healing because its direction is the same as what is observed in the endogenous EF (see Figure 3(b)) [2, 42–44]. Figures 4(c) and 4(d) show the 2D and 1D EF distributions, respectively, demonstrating a maximum EF of around 40 mV/mm near the edge of the wound (*x* = 8 mm and *x* = 12 mm).

**Figure 5:**
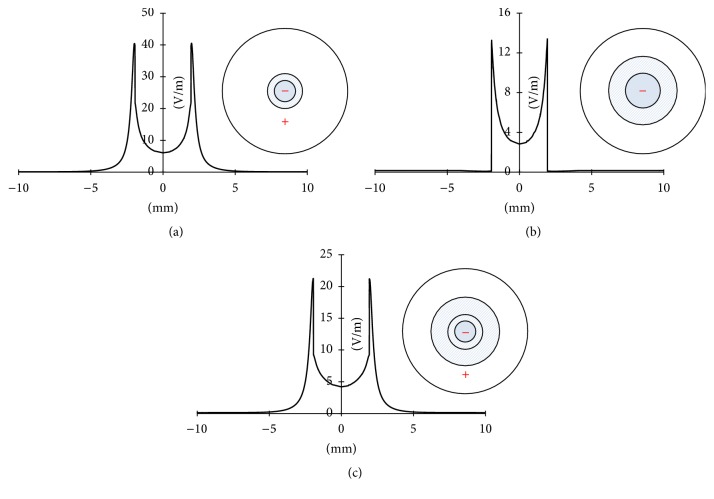
1D exogenous EF distributions in (a) Geo 2, (b) Geo 3, and (c) Geo 4.

Figures 5(a)–5(c) show the 1D exogenous EF distributions of the Geo 2, Geo 3, and Geo 4 configurations, respectively. In these three configurations, the directions of the electric current flows are similar to that observed in Geo 1, being a clockwise current flow outside the skin and a counterclockwise current flow inside the skin. Therefore, comparing with Figure 3(b), these exogenous EFs could have synergistic effects to the existing endogenous EF. In Geo 2, the maximum EF strength, around 40 mV/mm, occurred near the edge of the wound (*x* = 8 mm and *x* = 12 mm). This value is close to what is observed in Geo 1. However, in Geo 2, the EF decreased gradually outward along the intact skin (*x* < 8 mm and *x* > 12 mm) and reached zero on the outmost region (*x* < 3 mm and *x* > 17 mm). In Geo 3, the EF reached a maximum of around 13 mV/mm near the edge of the wound and then decreased to zero right out of the wound toward to intact skin. The EF distribution in Geo 4 is similar to what is observed in Geo 2, except that the EF had a maximum of only about 22 mV/mm. Figure  S1 of the Supplementary Material shows the 1D EF distributions combing the endogenous EF with the applied EF in different electrode configurations (see Supplementary Material available online at https://doi.org/10.1155/2017/5289041). Comparing all these configurations, we concluded that (1) all configurations resulted in the same electric current flow directions, which are helpful in electrotherapy applications, and (2) Geo 1 and Geo 2 provided the highest EF strength, compared to Geo 3 and Geo 4, indicating that covering the whole unwounded area (intact skin) with the positive electrode and the whole or part of the wounded area with the negative electrode resulted in the optimal configuration in such applications. The 1D EF distribution combining the endogenous EF with the applied EF in different electrode configurations is shown in Figure S1 of the Supplementary Material.

**Figure 6:**
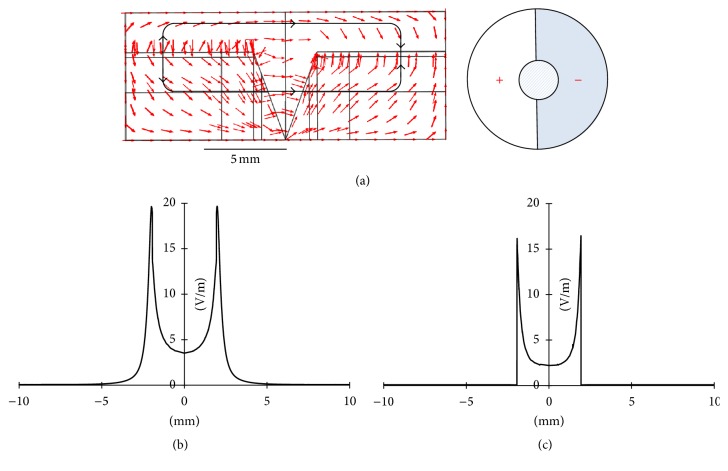
(a) Direction of the electric current flow in Geo 5 (Ambient = PBS). (b) 1D exogenous EF distribution in Geo 5 (Ambient = PBS). (c) 1D exogenous EF distribution in Geo 5 (Ambient = air).

In Geo 5, with the positive and negative electrodes being placed on each side of the intact skin, the directions of the electric current flow were opposite outside and inside the skin, as indicated in Figure 6(a) (see the black loop with arrows). A clockwise current flow was established outside the skin, and a counterclockwise one was observed inside the skin. As shown in Figure 6(b), the EF reached a maximum of about 20 mV/mm near the edge of the wound (*x* = 8 mm and *x* = 12 mm) and decreased gradually to zero outward along the intact skin (*x* < 8 mm and *x* > 12 mm). Clearly, this configuration was not suitable for electrotherapy applications because in the endogenous EF and other exogenous EFs (Geo 1~Geo 4) there are two current loops distributed symmetrically on each side of the wound but in the current configuration (Geo 5) there is only one current loop. After changing the surrounding medium from PBS to air, the difference in the EF distributions was significant. As shown in Figure 6(c), the EF strength was the highest in the edge of the wound (*x* = 8 mm and* x* = 12 mm) and was almost zero right outside of that edge along the intact skin (*x* < 8 mm and* x* > 12 mm). Moreover, the maximum EF strength was only around 16 mV/mm. This was simply due to the poor conductance of air, so it would be most helpful to keep the wound in moist, salty surroundings. This agrees with clinical findings that (1) cells die when they dry and (2) endogenous and exogenously enhanced electrotaxis is enhanced in a physiological moist wound environment.

Finally, we investigated the power dissipation density (in W/m^3^) due to Joule heating in different skin layers, as listed in Table 2. In the case of the endogenous EF, the power dissipation density in the stratum corneum was 8.9 W/m^3^, and this value decreased to 6.77 W/m^3^ in the epidermis, to 4.75 W/m^3^ in the dermis, and to 0.28 W/m^3^ in the subcutis. In Geo 1, these values were 8.05, 2.14, 2.34, and 0.12 W/m^3^ in the stratum corneum, the epidermis, the dermis, and the subcutis, respectively. In Geo 2~Geo 4, these values were 6.02~8.5, 0.39~1.54, 0.64~1.82, and 0.03~0.07 W/m^3^ in the stratum corneum, the epidermis, the dermis, and the subcutis, respectively. In the last configuration (Geo 5), the power dissipation densities were smaller compared to those in other cases, being 1.48 W/m^3^ in the stratum corneum, 0.36 W/m^3^ in the epidermis, 0.13 W/m^3^ in the dermis, and 0.02 W/m^3^ in the subcutis. These results suggested that most of the electrical energy was dissipated in the stratum corneum since it is the thinnest layer and has the smallest conductivity. According to [45], an absorption of surface power density less than 40 mW/cm^2^ was considered safe to the skin. In our studies, a power dissipation density of 10 W/m^3^ corresponded to only 1.4 × 10^−5^ mW/cm^2^ surface power density in the 0.014-mm-thick epidermis. Therefore, these electrotherapies with an applied voltage of 30 mV were thought to be harmless to the skin.

## 4. Conclusion

Knowing that the endogenous EF is beneficial and necessary for wound-healing, we built a three-dimensional wound model consisting of different tissue types in the skin layers to study the effects of electrode configurations, including sizes and positions, on the exogenous EFs produced around the wound. According to the results, different electrode configurations resulted in different magnitudes and distributions of exogenous EFs. The optimal arrangements were to cover the whole intact skin with the positive electrode and the whole or part of the wounded area with the negative electrode. With a potential difference of 30 mV established between positive and negative electrodes, these optimal configurations exhibited a maximum EF of around 40 mV/mm near the edge of the wound, which could have synergistic effects to the existing endogenous EF. The results also indicated that it would be helpful to keep the wound in moist, salty surroundings, comparing to the dry environment. Finally, by investigating the power dissipation density due to Joule heating in different skin layers, it was concluded that these different electrode configurations with an applied voltage of 30 mV should be harmless to the skin. The present study is beneficial to designing the electrode configuration for applications in clinical electrotherapies.

## Supplementary Material

Supplementary Figure S1: 1D EF distribution combing the endogenous EF with the applied EF in A. Geo 1, B. Geo 2, C. Geo 3, and D. Geo 4.

## Figures and Tables

**Table 1:** Parameters used in the wound model.

	Thickness (mm)	Conductivity (*σ* in Sm^−1^)	Relative permittivity (*ε*_*r*_)
PBS		1.4	80

Stratum corneum	0.014	2 × 10^−6^	5 × 10^2^
Epidermis	0.3	0.026	10^6^
Dermis	2.2	0.222	10^8^
Subcutis	3.0	0.08	10^7^

**Table 2:** Power dissipation densities (W/m^3^) in different configurations.

	Endo. EF	Geo 1	Geo 2	Geo 3	Geo 4	Geo 5
EF (mV/mm)	96	40	40	13	22	20

Stratum corneum	8.90	8.05	8.50	7.57	6.02	1.48
Epidermis	6.77	2.14	1.54	1.10	0.39	0.36
Dermis	4.75	2.34	1.82	1.16	0.64	0.13
Subcutis	0.28	0.12	0.07	0.04	0.03	0.02
