# Supplementary material for: Electrical Stimulation for Wound-Healing: Simulation on the Effect of Electrode Configurations
Source: Biomed Res Int. 2017 Apr 9;2017:5289041. doi: 10.1155/2017/5289041 (PMC5401728; doi:10.1155/2017/5289041)

Supplementary Material

The Supplementary Material including 1 supplementary figure is as follows.

Supplementary Figure S1: 1D EF distribution combing the endogenous EF with the applied EF in A. Geo 1, B. Geo 2, C. Geo 3, and D. Geo 4.

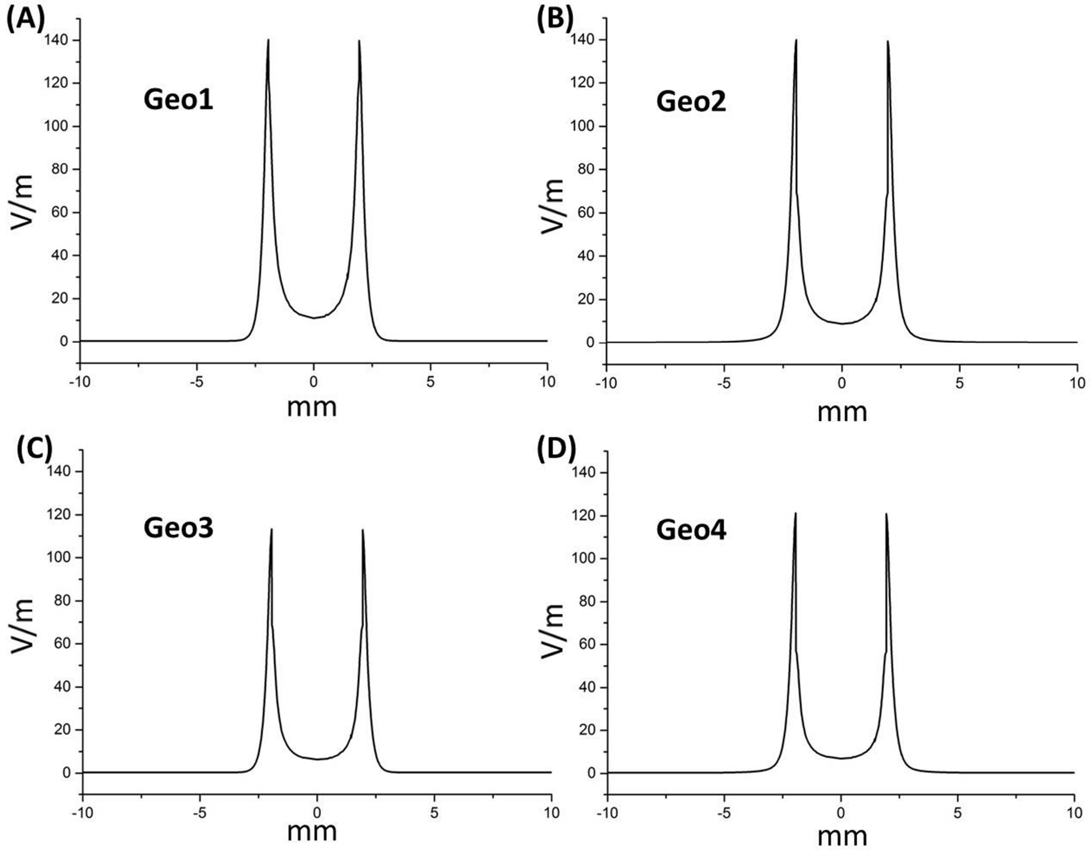

Supplement: Supplementary file 1 — Supplementary Figure S1: 1D EF distribution combing the endogenous EF with the applied EF in A. Geo 1, B. Geo 2, C. Geo 3, and D. Geo 4. [file 5289041.f1.pdf]
